# Selection of binding targets in parasites using phage-display and aptamer libraries *in vivo* and *in vitro*

**DOI:** 10.3389/fimmu.2012.00419

**Published:** 2013-01-09

**Authors:** R. R. Tonelli, W. Colli, M. J. M. Alves

**Affiliations:** ^1^Departamento de Ciências Biológicas, Universidade Federal de São PauloSão Paulo, Brazil; ^2^Departamento de Bioquímica, Instituto de Química, Universidade de São PauloSão Paulo, Brazil

**Keywords:** combinatorial methods for diagnosis and therapy, phage display, aptamers, SELEX, Kinetoplastidae, apicomplexa

## Abstract

Parasite infections are largely dependent on interactions between pathogen and different host cell populations to guarantee a successful infectious process. This is particularly true for obligatory intracellular parasites as *Plasmodium*, *Toxoplasma*, and *Leishmania*, to name a few. Adhesion to and entry into the cell are essential steps requiring specific parasite and host cell molecules. The large amount of possible involved molecules poses additional difficulties for their identification by the classical biochemical approaches. In this respect, the search for alternative techniques should be pursued. Among them two powerful methodologies can be employed, both relying upon the construction of highly diverse combinatorial libraries of peptides or oligonucleotides that randomly bind with high affinity to targets on the cell surface and are selectively displaced by putative ligands. These are, respectively, the peptide-based phage display and the oligonucleotide-based aptamer techniques. The phage display technique has been extensively employed for the identification of novel ligands *in vitro* and *in vivo* in different areas such as cancer, vaccine development, and epitope mapping. Particularly, phage display has been employed in the investigation of pathogen–host interactions. Although this methodology has been used for some parasites with encouraging results, in trypanosomatids its use is, as yet, scanty. RNA and DNA aptamers, developed by the SELEX process (Systematic Evolution of Ligands by Exponential Enrichment), were described over two decades ago and since then contributed to a large number of structured nucleic acids for diagnostic or therapeutic purposes or for the understanding of the cell biology. Similarly to the phage display technique scarce use of the SELEX process has been used in the probing of parasite–host interaction. In this review, an overall survey on the use of both phage display and aptamer technologies in different pathogenic organisms will be discussed. Using these techniques, recent results on the interaction of *Trypanosoma cruzi* with the host will be highlighted focusing on members of the 85 kDa protein family, a subset of the gp85/TS superfamily.

## Introduction

**Figure 1:**
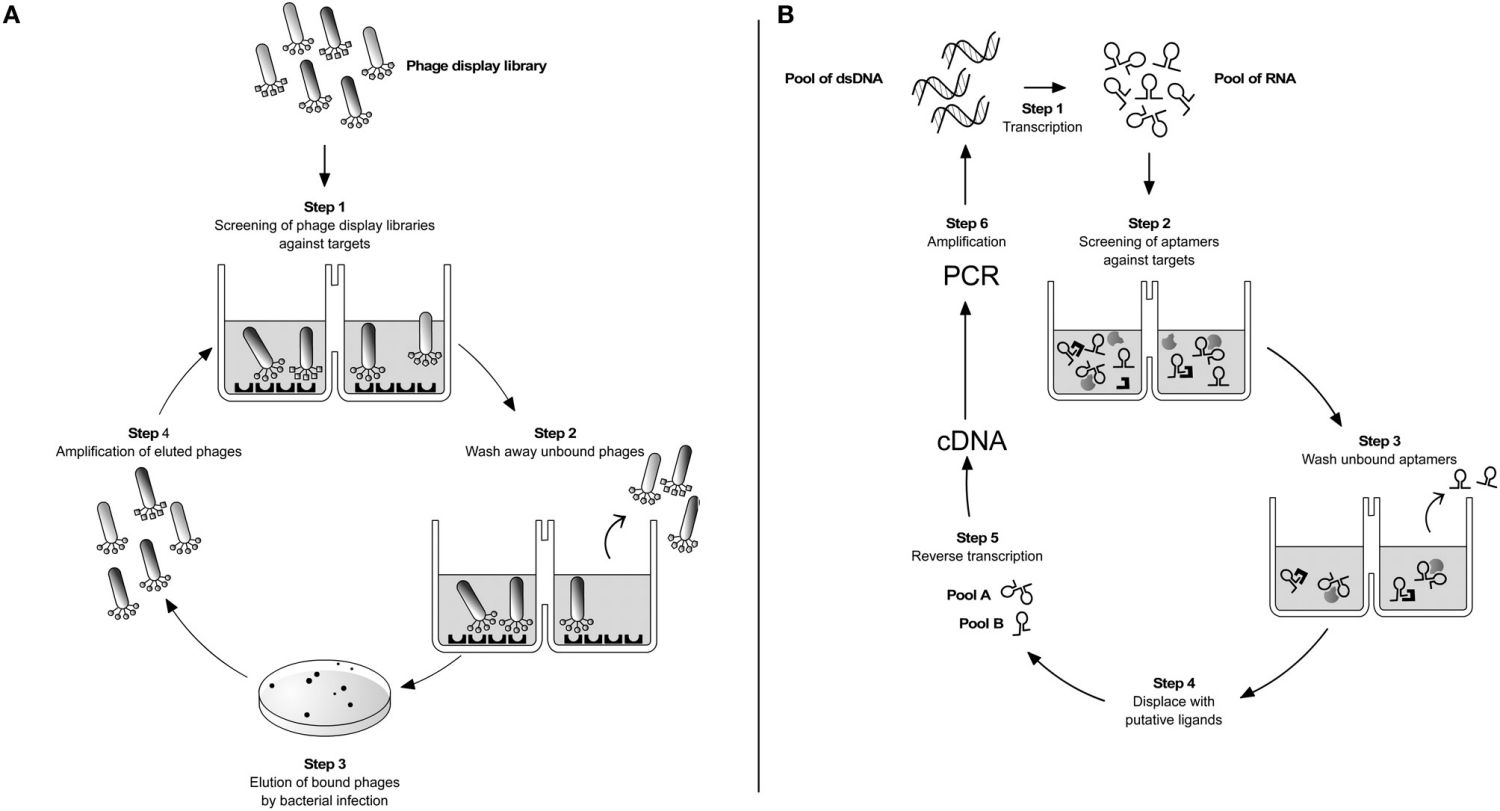
**Parasite targetting by combinatorial techniques. (A)** Phage display. A phage library displaying potential ligand proteins on their surface is exposed to an immobilized target (step 1). After washing away unbound phages (step 2), binders are eluted by *Escherichia coli* infection and plated on LB-agar (step 3). Clones are then amplified producing a phage mixture that is enriched with relevant (i.e., binding) phage (step 4). The repeated cycling of these steps is referred as “panning”. At the end of 3–4 rounds of panning the enriched phage population is recovered by infection of a suitable bacterial host and sequenced to identify the interacting peptides or protein fragments. **(B)** SELEX. It is based on a stretch of single-stranded nucleic acid, which can be RNA or single-stranded DNA (ss-DNA). These are chemically synthesized to have a random stretch usually from 8 to 40 nucleotides, flanked by constant sequences. In the case of RNA SELEX, the synthetic DNA template is transcribed into a pool of 10^13^–10^14^ different RNA molecules (step 1). The pool is incubated with the desired targets and due to the sample diversity some of the aptamers will bind to their targets (step 2). After washing out unbound RNAs (step 3) the different RNA pools are displaced by incubation with ligands of interest (step 4). By reverse transcription (step 5) and PCR amplification (step 6) selected double-stranded DNAs are reconstructed. The same cycle is repeated over 8–12 times until purified sequences specific for a given ligand are selected. The DNAs are cloned and sequenced. This iterative method follows the same logic when single-stranded DNA sequences are used as aptamers instead of RNA (Ulrich and Wrenger, 2009).

Phage display technology and RNA and DNA aptamers, developed by the SELEX process (Systematic Evolution of Ligands by Exponential enrichment), were described over two decades ago and since then contributed to a large number of small peptides or structured nucleic acids for diagnostic or therapeutic purposes or for the understanding of the cell biology (Figure 1). Comparison of normal cells with cancer or infected cells and vascular endothelium of organs are the most visible examples. The small peptides or nucleic acids of interest can be modified to increase their half-life in the organism or to be conjugated to other molecules, such as fluorescence probes, nanoparticles, or immobilized matrices, increasing their potential use.

## Phage display

The use of filamentous bacteriophages (virus that infect bacteria) to express and display foreign protein fragments or peptides started in the mid-1980s when a portion of the gene encoding the *EcoRI* endonuclease was fused to the gene coding for the pIII protein coat from a M13 virus (Smith, 1985). The result of this original experiment was the production of hybrid filamentous bacteriophages, or fusion phages, expressing and displaying the product of the fusion gene into the minor capsid protein pIII on the surface of the phage particle (Smith, 1985). This approach represented a landmark in the field of molecular genetics, because firstly foreign DNA was directly linked to the replicating phage genome and secondly, the phage operated like an “expression vector” with the foreign DNA being expressed as a “protein” associated to the phage protein coat (Smith and Scott, 1993; Smith and Petrenko, 1997).

Most phage-display work has used filamentous phage strains (M13 and its close relatives fd and f1) as vectors. Filamentous bacteriophage consists of a circular single-stranded DNA (ssDNA) genome covered by a few thousand copies of the major coat protein pVIII (~2700 copies in wild type phage) with each end capped by five copies of two different sets of proteins: pIII and pVI at the end that binds to bacteria and inject the DNA into the host cell; pVII and pIX at the other end (Marvin, 1998). Each of the five capsidic proteins has been used to display foreign polypeptides on the surface of the M13 bacteriophage but the minor protein pIII is most commonly used (Greenwood et al., 1991; Smith and Petrenko, 1997). Despite the ease manipulation and extraordinary stability of the phage particle, the display of foreign proteins is not without difficulties and imposes that polypeptides have a limited size, sequence, and folding characteristics, as large molecules may compromise the structure and function of the protein coat (Sidhu, 2000). This restraint has led researchers to cast about for new protein display scaffolds resulting in the development of phagemid display systems (Qi et al., 2012). Phagemid was developed as a hybrid of the filamentous phage M13 and a plasmid to produce a vector that can grow as a plasmid, and also be packaged as single stranded DNA in viral particles. When introduced into a bacterial host together with a “helper-phage,” phagemid systems allow the display of both fusion and wild type coat proteins attenuating possible defects on phage function (Sidhu, 2000; Qi et al., 2012).

In recent years display of heterologous proteins on the surface of microorganisms is not restricted to the filamentous phage (Smith, 1985). Alternative display systems that use bacteriophage λ (Sternberg and Hoess, 1995), lytic phages like T4 and T7 (Efimov et al., 1995; Ren et al., 1996), eukaryotic viruses such as baculovirus (for a review see Makela et al., 2010), bacteria (Georgiou et al., 1997), and yeast are also used, each one having advantages and disadvantages with respect to each particular application.

The benefits behind the display systems have also encouraged the development of a new generation of selection technologies. Cell-free systems like ribosome (He and Taussig, 2002), mRNA (Takahashi et al., 2003) and DNA display technologies (Yonezawa et al., 2004) are proven to be more advantageous than phage display, as larger libraries (up to 10^14^) can be constructed. While in the ribosome display, individual nascent proteins are coupled to their corresponding mRNA through the formation of stable ternary protein–ribosome–mRNA (PRM) complexes, in the other two systems protein fragments and peptides are covalently coupled to a DNA/RNA template (He and Taussig, 2002; Takahashi et al., 2003; Bertschinger and Neri, 2004). These technologies are becoming more commonplace, but the phage display platform remains the leading technology (Sidhu, 2000).

Phage display libraries are heterogeneous mixtures of fusion phages, each one carrying a different foreign DNA insert and displaying a different protein on filamentous phage surface (Cwirla et al., 1990; Devlin et al., 1990; Scott and Smith, 1990; Smith and Petrenko, 1997). Such libraries containing billions of phage clones (some libraries are as high as 10^12^ diverse) are extensively used to screen and select for peptides that bind with high-affinity to target molecules. The selection procedure known as “panning” or “biopanning” (depending whether selection is performed *in vitro* or *in vivo*, respectively) is simple and involves four major steps (Figure 1) (Parmley and Smith, 1988). After 3–5 rounds of selection against the desired target, individual clones are isolated and the primary structure of the binding peptides is deduced by nucleotide sequencing. Peptide sequences obtained in this manner may then be used in alignment search for known proteins (Koivunen et al., 1999).

### Applications of phage display

One of the earliest applications of phage display technology was to study antigen–antibody binding for the identification of epitopes and mimotopes (small peptides that mimic linear, discontinuous, and even non-peptide epitopes) (Germaschewski and Murray, 1995). However, it was later shown that larger molecules like antibody fragments (scFV, Fab fragment, VHH domains) could be successfully displayed on phage (McCafferty et al., 1990; Benhar, 2001; Bradbury and Marks, 2004; Petrenko, 2008). The development of phages that display antibodies led to the emergence of a new molecular recognition interface to study protein–protein interactions, structure-function relationships, and protein folding and stability (Clackson et al., 1991; Vaughan et al., 1996). As a result, phage displayed cDNA libraries advanced significantly with a plethora of ligands fused to phage particles such as glycoproteins (Celik et al., 2010), enzymes (Soumillion et al., 1994), protease inhibitors (Markland et al., 1996), cytokines (Gram et al., 1993; Buchli et al., 1997), secreted as well as cytoplasmic, nuclear and membrane proteins (Vithayathil et al., 2011) to cite a few examples.

With the increasing number of phage display collections, numerous new applications have emerged. Selections from various libraries have been used to identify peptide agonists and antagonists for receptors (Sidhu, 2000), determine bind specificity of domains (Sparks et al., 1996; Linn et al., 1997), map carbohydrates and protein functional epitopes (Sidhu, 2000; Fukuda, 2012), select antibodies recognizing post-translational modifications (Kehoe et al., 2006), identify targets for the inhibition of tumor-specific angiogenesis (Koivunen et al., 1999; Arap et al., 2002; Zurita et al., 2003), vaccine development (Lidqvist et al., 2008), and molecular imaging with the use of fluorescently labeled phage (Newton et al., 2006; Petrenko, 2008). More recently, there is a strong trend for the use of phage display in medical science with the production of humanized antibodies and development of new therapeutics. Indeed, a few selected peptides and proteins are in clinical or preclinical stages of development and some are reaching the market (Rothe et al., 2006).

### The use of phage display in the study of parasitic infections

Infectious diseases may be caused by various pathogens like bacteria, fungi, virus, multi and unicellular parasites. Independently of the mechanism by which the pathogenic agent elicits infection, the surface components are key determinants for the disease progression. Over the years several laboratories have made great effort to elucidate the mechanisms involved in the establishment of infectious diseases and not surprisingly many of them have focused on the discovery of molecules, from both the pathogen and the host. Considering this, much of the work on pathogen–host interaction relied on classical biochemical and cellular biology approaches (purification, cross-linking, immunoprecipitation, and fractionation) to track for ligands and receptors. Nevertheless, with the rapid expansion of the phage display platform and the development of high throughput selections, pathogenic agents and their hosts are currently being mapped for toxins, ligands and interacting proteins as well for the identification of possible drug candidates.

### Studies on apicomplexa using phage display

#### Plasmodium

The phylum apicomplexa encompasses a vast array of organisms, some of which are of major importance in veterinary and medical areas. Members of the genus *Plasmodium*, *Toxoplasma* and *Cryptosporidium*, for example, are pathogens of humans and cause, respectively, malaria, toxoplasmosis and cryptosporidiosis. The genus *Eimeria* contains important parasites of livestocks, especially poultry, causing coccidiosis. All are unicellular eukaryotes and are obligatory intracellular parasites circulating between an intermediate and a definitive host. The potential of the phage display system in the field of parasitology was first demonstrated in studies on malaria.

*Plasmodium* protozoa are transmitted to humans by the bite of female mosquitoes from the Anopheles genus. Infection in humans begins when the sporozoites, injected with the saliva of the mosquito vector during blood meal, spreads through the bloodstream reaching the liver. Once inside the hepatocytes (exoerythrocytic cycle), sporozoites divide repeatedly and differentiate into merozoites, which once released in the bloodstream infect the red blood cells (RBCs) beginning the intraerythrocytic cycle. The surface of RBCs is covered by sialoglycoproteins from which glycophorins are the major constituent being implicated in merozoite invasion (Howard et al., 1982). To identify the molecular ligands for the glycophorins, a phage display cDNA library from a *P. falciparum* strain dependent on sialic acid to invade RBCs was used in panning assays against purified human MN-glycophorins or isolated RBCs. After four panning rounds, sequencing of individual positive clones and BLAST searches using the PlasmoDB, several parasite proteins that specifically bind to glycophorins and RBCs were identified. Among them were the erythrocyte-binding antigen (EBA-175) and the erythrocyte-binding ligand-1 or EBL-1, both belonging to the superfamily of the erythrocyte binding-like (EBL) proteins (Li et al., 2012). In addition, the phage cDNA insert bearing the binding site for glycophorin encoded a 69-amino acid peptide located within a subdomain (D2) of the Duffy binding-like domain (DBL) of the EBL-1 gene (Li et al., 2012).

A different strategy to study host–parasite interactions during the intraerythrocytic life cycle of *Plasmodium* was the use of a peptide phage display library to identify peptides with affinity for invading merozoites and infected RBCs (iRBCs). When merozoites are prone to invade the erythrocytes, they translocate and expose on their surface a type I integral membrane protein known as the apical membrane antigen-1 (AMA-1) (Triglia et al., 2000). This protein undergoes proteolytic cleavage at around the point of host invasion and this processing is essential for parasite entry as antibodies that prevent AMA-1 proteolysis inhibit RBC invasion *in vitro* (Dutta et al., 2003). Moreover, during the development of merozoites inside the RBCs, *Plasmodium* proteins are associated or inserted into the host plasma membrane dramatically altering the composition and structure of the erythrocyte membrane. This event is an important mechanism exploited by *Plasmodium* to evade the host immune system since erythrocytes become covered with variant antigens (antigenic variation) protecting the parasite from variant-specific antibodies elicited by earlier infections. This variation is mediated by the differential control of a family of surface molecules termed PfEMP1 and encoded by approximately 60 *var* genes as described by the genome sequencing of *P. falciparum* (Aley et al., 1984; Gardner et al., 2002). Considering these data, panning of random peptide phage display libraries against AMA-1 and the altered surface of infected *Plasmodium* erythrocytes were performed to identify mimotopes for merozoites and iRBCs. One of the peptides that bound to AMA-1 was shown to be a potent inhibitor of the invasion of *P. falciparum* merozoites into human erythrocytes (Li et al., 2002). When the targets were the iRBCs, one selected peptide (P1) that constituted more than 40% of the sequenced phage clones bound specifically to the surface of infected erythrocytes and showed anti-malarial activity (Eda et al., 2004).

Phage display has also been used to investigate the host immunological response against the sporozoites in the pre-erythrocytic cycle. Peptides that represented the circumsporozoite protein (CSP), the major surface antigen of the sporozoites, were inserted into the gene of PVIII of filamentous phage and used to immunize rabbits to test for the antigenicity of CSP epitopes. These experiments have shown that CSP can generate a protective antibody response in addition to provide insights into new antigenic epitopes for vaccine development against malaria parasites (Greenwood et al., 1991).

In an effort to control the development of *Plasmodium* inside the invertebrate host, a work focused on the study of *Anopheles*-malaria parasites interaction was undertaken. Using a phage display library and successive rounds of biopanning with female mosquitoes, a single peptide (SM1) was selected that bound specifically to the luminal side of the midgut epithelium and to the distal lobe of the salivary glands inhibiting *Plasmodium* invasion of the two organs (Ghosh et al., 2001, 2002). Examples on the work of malaria research using phage display technology are listed in Table 1 (Lanzillotti and Coetzer, 2008).

**Table 1:** **Summary of phage display applications in malaria research**.

**Target**	**Library**	**Result**	**References**
Mosquito epithelia	Random peptides	Identification of SM1 peptide that inhibits salivary gland invasion by *Plasmodium*	Ghosh et al., 2001, 2002
Ookinete surface	Random peptides	Identification of enolase and actin on the surface of ookinetes	Hernandez-Romano et al., 2011
Parasitized erythrocyte	Random peptides	Identification of a peptide that binds to the surface of *Plasmodium*-infected erythrocytes. Isolation of an antibody against a peptide that causes hemolysis of iRBCs	Eda et al., 2004
Erythrocyte surface proteins	*P. falciparum* cDNA phage library	Identification of *Pf*EBL-1 that bind glycophorin B on the surface of erythrocytes	Li et al., 2012
Purified human erythrocyte protein 4.1	*Plasmodium* cDNA phage library	Identification of EBA-175, EBL-1 and a Ser/Thr kinase as ligands for protein 4.1	Lauterbach et al., 2003
AMA-1	Random peptides	Identification of peptides that bind AMA-1 and inhibit parasite invasion of erythrocytes; structure determination of AMA-1 epitope targets of inhibitory human antibodies	Nair et al., 2002; Keizer et al., 2003
	Mouse antibodies	Identification of four antibodies specific for *Plasmodium* AMA-1	Sabo et al., 2007
Anti-AMA-1 and anti-rhoptry mAbs	Random peptides	Identification and structural elucidation of AMA-1 mimics. Antibodies to mimotopes that inhibit erythrocyte invasion by *Plasmodium*	Narum et al., 2006
CSP	Phage library of CSP peptides	Induction of a strong and specific immune response. Structure determination of a CSP epitope	Greenwood et al., 1991; Monette et al., 2001
MSP	Human and mouse antibodies	Isolation of three antibodies against MSP-3 and MSP-1 that inhibit parasite growth	Sowa et al., 2001; Lundquist et al., 2006; Cheng et al., 2007
	Random peptides	Identification of MSP mimotopes	Demangel et al., 1996
SERA5 enzyme	Random peptides	Identification of a peptide that binds the catalytic domain of the enzyme and affects intraerythrocytic development	Fairlie et al., 2008
Pfs48/45 protein	Human antibodies from malaria-immune patients	Isolation of a scFV that reacts with sexual stages of *P. falciparum* and Pfs48/45 protein	Roeffen et al., 2001
Duffy binding protein (DBP)	Human antibodies from malaria-immune patients	Identification of three scFV that binds (DBP) inhibiting adhesion of RBCs	Kim et al., 2007

#### Toxoplasma gondii

*Toxoplasma* parasites are another good example of apicomplexa that served as excellent models in studies involving phage display technology. Infection with *T. gondii* occurs by the ingestion of either infectious oocysts through contact with cats (the definitive host) or cat feces or by feeding with meat that contains tissue cysts. Although opportunistic in healthy humans, toxoplasmosis is a lethal disease when progressing in chronically infected individual developing immunodeficiency, particularly in AIDS patients. As a consequence, much of the work on *Toxoplasma* and phage display is focused to cover different aspects of the immunological response against this parasite. To identify antigens implicated in human B-cell responses, a lambda phage-display library of *T*. *gondii* cDNA fragments was screened with sera of infected individuals. As a result, recombinant phage clones were identified carrying B-cell epitopes present in different *T*. *gondii* antigens like SAG1, GRA1, GRA7, GRA8, and MIC5 (Beghetto et al., 2003). Using a similar strategy, this group screened a panel of sera of pregnant women infected with *T. gondii* and identified an antigenic peptide within the sequence of the dense granule GRA1 protein (p24) that is immunodominant in *Toxoplasma* infections (Beghetto et al., 2001). Altogether, these data demonstrated the potential of phage-display technology for antigen discovery and for the study of the human antibody response on infectious agents. Data in Table 2 summarize the work on *Toxoplasma* research using phage display technology.

**Table 2:** **Summary of phage display applications in *T. gondii* research**.

**Target**	**Library**	**Result**	**References**
Sera of *T. gondii*-infected individuals	*T. gondii* cDNA phage library	Identification of epitopes of the *T. gondii* antigens	Beghetto et al., 2003; Di Cristina et al., 2004
Sera of *T. gondii*-infected pregnant women	*T. gondii* cDNA phage library	Identification of an epitope GRA1 protein	Beghetto et al., 2001
Anti-*T. gondii* mAbs	*T. gondii* cDNA phage library	Identification of an epitope of GRA3 located in the dense granules of *T. gondii* tachyzoites	Robben et al., 2002
	Random peptides	Identification of a linear epitope within SAG2A that is expressed in *T. gondii* tachyzoite surface	Cunha-Junior et al., 2010
TgMIC2	Mouse antibodies from *T. gondii* immune animals	Identification of scFV antibodies that recognize TgMIC2	Hoe et al., 2005

#### Cryptosporidium parvum

To date, 20 different species of *Cryptosporidium* have been described based on differences on the host species they infect. *Cryptosporidium* species are enteric coccidia parasites causing cryptosporidiosis (an acute, non-bloody, watery-diarrhea) in mammalian hosts. Table 3 summarizes work on *Cryptosporidium* using phage display technology. *C. parvum* is of particular concern as it infects humans, especially immune compromised individuals where the disease may become chronic and cause a life-threatening gastroenteritis with a high mortality. Infection with *C. parvum* occurs when ingested oocysts release sporozoites in the intestines of a suitable host. The sporozoites then attach to and invade epithelial cells of the gastrointestinal tract where they undergo intracellular development through asexual as well as sexual cycles. Even though many proteins, such as GP900, CP47, TRAP-C1, GP40, and GP15, have been implicated in the infection, increasing knowledge on the host–parasite interacting molecules is crucial for designing strategies to combat cryptosporidiosis. This has been investigated by panning a cDNA library of the sporozoite and oocyst stages of *C*. *parvum* expressed on the surface of T7 phage against intestinal epithelial cells (IECs). This study has identified a surface 12 kDa protein (CP12) that localizes especially at the apical region of sporozoites (Yao et al., 2007). Another work has identified the CP2, a known surface molecule of sporozoites involved in the invasion process, when the *C. parvum* T7 phage display library was screened by using Caco-2 (human epithelial colorectal adenocarcinoma) cells (Guo et al., 2009). Single-chain variable fragment (scFv) phagemid library has also being used to identify for specific scFvs that bind to *C. parvum*. As a result, panning against the surface protein P23, revealed two scFv sequences that were able to detect both native *C. parvum* proteins and recombinant P23. Importantly, the selected fragments showed no cross-reactivity with *E. coli*, *S. pyogenes*, *L. monocytogenes*, *B. cereus*, *G. lamblia* (cysts or trophozoites), or with S16, another dominant surface antigen on *C. parvum* sporozoites (Boulter-Bitzer et al., 2009).

**Table 3:** **Summary of phage display applications in *C. parvum* research**.

**Target**	**Library**	**Result**	**References**
*C. parvum* glycoproteins	*C. parvum*-specific polyclonal antibody library	Identification of antibodies that recognize *C. parvum* glycoprotein and oocyst/sporozoite preparations	Chen et al., 2003
*C. parvum* oocysts	Human semi-synthetic phage display antibody libraries	Isolation of scFv antibodies that block infection of HCT-8 cells by *C. parvum*	Pokorny et al., 2008
Sporozoite surface antigen S16 and P23	Human semi-synthetic phage display antibody libraries	Isolation and identification of scFV antibodies that bind to *C. parvum*	Boulter-Bitzer et al., 2009, 2010
Intestinal epithelial cells (IECs)	*C. parvum* cDNA phage library	Identification of a surface adherence protein (CP12) from sporozoites	Yao et al., 2007

#### Eimeria

Infection of chicken with ***Eimeria*,** the causative agents of coccidiosis, is a serious problem for the poultry industry being responsible for varying degrees of morbidity and mortality between infected birds. *Eimeria* parasites invade and develop within the avian IECs causing tissue damage that results in blood loss, dehydration, nutrient malabsorption, and increased susceptibility to other opportunistic pathogens. The parasite is acquired by the ingestion of sporulated oocysts that release sporocysts, which in turn release invasive sporozoites. These invade the avian intestinal tract and once inside the host cells they round up as a trophozoite. At the end of the cycle, *Eimeria* parasites leave the cells as merozoites, which further infect fresh host cells. Control of avian coccidia is being a main challenge in veterinary science and, consistently, studies using phage display are directed toward this end. This is reflected on a work where a phage display library was used to screen for peptides with *in vitro* activity against *E. acervulina* and *E*. *tenella* sporozoites using living purified *E*. *acervulina* sporozoites as targets. The selected peptide (PW2) disrupted the sporozoite pellicle, similarly to most natural antimicrobial peptides and showed a very low lytic effect on mammalian and avian cells, suggesting a potential use as a drug against avian coccidiosis. A variation on this theme is the panning of antibody libraries against different development of *Eimeria* parasites. Heavy chain (VH) and light chain (VL) antibody libraries against *E. acervulina* merozoites (Zhao et al., 2010) and scFV antibody libraries against *E. tenella* sporozoites (Abi-Ghanem et al., 2008) were used aiming at the development of new tools for diagnosis and therapy against coccidiosis. Selected examples of these applications are listed in Table 4.

**Table 4:** **Summary of phage display applications in *Eimeria* research**.

**Target**	**Library**	**Result**	**References**
*Eimeria* sporozoites/live	Random peptides	Identification of PW2 peptide with activity against *E*. *acervulina* and *E*. *tenella* sporozoites	da Silva et al., 2002
*E. tenella* sporozoites/cryopreserved	Chicken scFV antibodies from *E. tenella* immune birds	Isolation of an scFV antibody that binds specifically to *E. tenella* sporozoites	Abi-Ghanem et al., 2008
Cryopreserved *E. acervulina* merozoites	Chicken scFV antibodies from *E. tenella* immune birds	Identification of antibody fragments with high specificity and binding capacity for soluble antigens and intact fixed merozoites	Zhao et al., 2010
Anti-GAM56 protein	*E. tenella* cDNA phage display	Identification of EtGAM22 expressed predominantly at the gametocyte stage	Krucken et al., 2008

### Studies on kinetoplastidae–host interactions using phage display

The Kinetoplastidae are a class of unicellular and flagellated protozoan parasites responsible for serious diseases in humans and animals. Members of this class include all species of Trypanosomatidae, as *Leishmania* and *Trypanosoma* (*T. cruzi* and *T. brucei*).

#### Trypanosoma cruzi

*T. cruzi*, the American trypanosome, is an obligatory intracellular parasite that circulates between an invertebrate host (Triatomine insects) and a vertebrate host. In the latter, development of *T. cruzi* parasite begins with an intracellular amastigote form that reproduce within different cell types by binary fission. After successive rounds of replication, amastigotes differentiate into the infective and non-replicative trypomastigotes, which are released from the infected cell. The surface of the infective trypomastigotes of *T. cruzi* is covered by glycoproteins important for the adhesion and/or internalization of the parasite to host cells and one example is the superfamily of GPI-anchored glycoproteins named gp85/trans-sialidase. The majority of this superfamily members have at the subterminal carboxyl side the motif VTVxNVFLYNR (known as FLY domain) involved in host cell binding (Magdesian et al., 2001). It has long been known that heart and gastrointestinal tract cells are important targets for *T. cruzi* infection playing significant roles in pathogenesis and maintenance of parasitic reservoirs. The nature of this tropism is not well known and understanding the determinants of this feature can contribute to *T. cruzi* control and drug development. Interestingly, random peptide libraries of bacteriophages injected intravenously pointed out to differences in the blood vessels of different organs (Pasqualini and Ruoslahti, 1996; Arap et al., 2002), raising the notion that the vascular bed expressed specific molecular markers or ZIP codes allowing the delivery of cells or molecules to designated targets (Rajotte et al., 1998). Infectious agents could also exploit these differences in the expression of vascular addresses to gain access to target tissues/organs were they could develop. A combination of immortalized endothelial cells (ECs) and phage display methodologies were employed to investigate whether the FLY domain could interact with the blood vessel and contribute to *T. cruzi* tissue homing. Initially, filamentous bacteriophages were genetically engineered to display the FLY peptide on the pIII minor coat protein (FLY phage). Using that strategy, it was shown that the FLY phage, but not the control phages, binds strongly to ECs derived from the heart and the bladder, two organs in which parasites can be found following infection (Tonelli et al., 2010). Furthermore, hybrid phages injected intravenously into mice demonstrated that FLY binds to the vasculature of different organs but is enriched in the vasculature of the heart, bladder, and esophagus in agreement with the cell binding *in vitro* assay (Tonelli et al., 2010). These results indicated that *T. cruzi* tropism at the molecular level could be explained, at least in part, through the interaction of the FLY motif with receptors present in the vascular bed of different organs. Indeed, this was the first experimental demonstration on how the use of phage display technologies can help to elucidate the mechanisms by which *T. cruzi* interacts with blood vessel receptors *in vivo*.

A phage display library was employed to help the identification of B-cell epitopes on the *T. cruzi* trans-sialidase (TcTS) (Pitcovsky et al., 2001). Trans-sialidase, a GPI-anchored glycoprotein, is shed by *T. cruzi*, being detected in the blood of infected patients during the acute phase of the disease. This enzyme is responsible for the direct transfer of sialyl residues from the host cells to acceptor proteins on the surface of infective trypomastigotes. Both, phage display technology and affinity-purified TcTS antibodies from *T. cruzi* infected rabbits were instrumental to isolate several B-cell epitopes located within or near the catalytic N-terminal domain of TS showing reactivity with sera obtained from *T. cruzi* infections. More importantly, some of the selected peptides were exposed on the surface of the TcTS probably explaining their strong antigenicity (Pitcovsky et al., 2001). Using the same approach the trypomastigote-restricted shed acute-phase antigen (SAPA) from *T. cruzi* was also mapped. The SAPA antigen is a 12-amino acid repetitive unit displayed *in tandem* on the carboxyl-terminus of the TcTS and it is the immunodominant antigen during *T. cruzi* infections. Screening of a phage display library of random peptides against anti-SAPA mAbs and purified immunoglobulin G from SAPA-immunized rabbits resulted in the identification of multiple linear overlapping B-cell epitopes within the repeated unit of SAPA (Alvarez et al., 2001). These data shed light on the molecular structure of the SAPA antigen and on how repetitive antigens are recognized by the immune system. Table 5 summarizes work on trypanosomes.

**Table 5:** **Summary of phage display applications in research with *T. cruzi* and *T. brucei***.

**Target**	**Library**	**Result**	**References**
*T. cruzi*			
Mouse vascular bed	Hybrid phage displaying the FLY domain	FLY interacts with the endothelium in an organ-dependent manner with a preference for the heart vasculature	Tonelli et al., 2010
TcTS-specific antibodies and anti-SAPA mAbs	Random peptides	Identification of B-cell epitopes located on TcTS and the SAPA antigen	Pitcovsky et al., 2001
*T.b. gambiense*			
Sera of *T.b. gambiense*-infected mouse and humans	Random peptides	Identification of epitopes of the *T.b. gambiense* VSG antigens	Van Nieuwenhove et al., 2011, 2012

#### African trypanosomes

Sleeping sickness or human African trypanosomiasis (HAT) is caused by subspecies of the protozoan parasite *T. brucei (T. b. gambiense* in West and Central Africa). *T. brucei* is transmitted by tsetse flies of the genus Glossina and once inside the vertebrate it escapes from the host immune system by continuously replacing the major parasite antigens on the plasma membrane, the variant surface glycoproteins or VSG. This successful tactic of the parasite to avoid the defense of the host turned *T. brucei* detection laborious and insensitive, limiting the identification of possible infected individuals. This observation led investigators to search for new ways of accurately identifying HAT-specific VSG epitopes (mimotopes) possibly recognized by the host immune system. Two studies illustrate well this idea (Van Nieuwenhove et al., 2011, 2012). In one study, two phage display libraries of random peptides (12-mer and cyclic 7-mer) were screened with mouse MAbs against the two predominant *T.b. gambiense* VSGs proteins (LiTat 1.3 and LiTat 1.5). The result was the isolation of several peptides that mimicked epitopes on the native trypanosomal VSGs LiTat 1.5 and LiTat 1.3 (Van Nieuwenhove et al., 2011). Some of the selected peptides were then confirmed as mimotopes for the parasite VSGs since they were able to inhibit the binding of their homologous monoclonal to the corresponding VSG (Van Nieuwenhove et al., 2011). In a second work focused on the screening of phage display libraries against polyclonal antibodies from sleeping sickness patients sera, two mimotopes of VSG have been found to react positively in indirect ELISA with a panel of 102 HAT positive and 102 endemic negative sera (Van Nieuwenhove et al., 2012). Both studies illustrate how the phage display platform can help to isolate epitopes against infectious agents with potential use in diagnostics. Table 5 summarizes work on trypanosomes.

## Aptamers

The SELEX technology is an oligonucleotide-based combinatorial library method to select high affinity ligands (aptamers, from aptus, “to fit”) to almost any target molecule, including peptides, or cells (Cell-SELEX) (Ulrich and Wrenger, 2009; Ahmadvand et al., 2011; Gold et al., 2012). It is based on a stretch of single-stranded nucleic acid, which can be RNA or ssDNA. These are chemically synthesized to have a segment usually from 8 to 40 nucleotides randomly bound. These sequences tend to assume different conformations, due to the formation of: (1) hairpins, which are helical double stranded structures due to complementarity of nucleotides, like in the classical t-RNA secondary structures; (2) G-tetrads, which is an association of four guanine bases through hydrogen bonding forming a planar squared structure; when these tetrads pile on top of each other and are stabilized by a cation, a G-quadruplex is formed (Johnson et al., 2008); (3) bulges, which occur when a duplex helix is interrupted by a single stranded stretch of nucleotides in only one strand; usually this may form a weak point leading to bending of the helical axis (Lilley, 1995); and (4) pseudo-knots formed by the intercalation of stem-loop structures (Staple and Butcher, 2005). These different topologies with varying loop configurations change the whole geometry of the nucleic acid molecules determining the formation of secondary and tertiary structures that provide a large diversity for the recognition of specific molecules and receptors on cell surfaces. Thus, aptamers are bioactive target-specific compounds that can be used to identify and modulate the activity of surface targets embedded in the membrane of cells relevant for human disease, either for diagnostic or therapeutic purposes.

The SELEX technique uses reiterative *in vitro* selection of combinatorial RNA or DNA pools against a target molecule for the identification of aptamers that are high-affinity oligonucleotide ligands. Aptamers isolated by such iterative method recognize their targets with binding specificities and affinities comparable to those of monoclonal antibodies, with the advantage to be easily chemically modified as to avoid nuclease attack. Thus, due to the fact that aptamers may recognize epitopes exclusively expressed by the target cell, they may be employed even to recognize different physiological states of a given cell, being capable, for example, to distinguish between protein isoforms and different conformations of the same protein.

When RNA aptamers are desired, the RNA pool used in the selections is transcribed from a pool of synthetic DNA templates (Figure 1). Each of these may contain, for example, 108 nucleotides with a 40 nucleotide randomized region flanked on both sides by constant sequences. The 5′ nucleotide sequence upstream the randomized region is a promoter for T7 phage RNA polymerase (Famulok et al., 2000). Two-fluoro-pyrimidine triphosphates can be used as substrates for RNA polymerase to provide stability of the transcribed products against nuclease attack (Ito et al., 1998). Such synthetic DNA pool is transcribed using T7 RNA polymerase in the presence of 2′-OH-ATP and GTP and 2′-F-CTP and UTP (Ruckman et al., 1998).

The obtained RNA molecules can now be incubated with any target, a protozoon for example, to enable RNA:cell surface binding to occur (cell-SELEX). The cell:RNA complexes are separated from free RNA molecules and the bound RNA is eluted by competition with the desired ligand. The liberated RNA is then isolated, reverse transcribed, and amplified by standard PCR procedures (Figure 1). This SELEX procedure will be repeated for 9–12 selection rounds until no further increase in binding affinity can be measured (Irvine et al., 1991). To obtain more specificity, one or more of the iterative rounds can be done against a cell close to the one of interest in order to select out aptamers that are shared by different differentiation forms (negative selection). For example, one may use merozoites of *P. falciparum* to eliminate RNAs bound to targets that are shared with sporozoites. The sequence described above follows the same logic when aptamers carrying ssDNA sequences are used instead of RNA (Ulrich and Wrenger, 2009).

Targets can be isolated either by aptamer immobilization on magnetic beads or by affinity chromatography. Also, receptors on cells can be photocrosslinked with their RNA/DNA aptamers to facilitate target protein isolation. Finally, aptamers can be labeled with fluorescent labels to facilitate separations of cells by FACS (Ulrich and Wrenger, 2009).

SOMAmers (Slow Off-rate Modified Aptamers), a term introduced by Gold et al. (2010), are short, single stranded deoxyribonucleotides, bearing dU-modified residues. Like aptamers, they can be selected against any target molecule. The method for selecting SOMAmers with low dissociation rates (t_1/2_ > 30 min) and elimination of non-specific binding (employing an excess of a polyanionic competitor, for example) were developed and reported to show high success rates on selection when compared to the traditional aptamers: using four modified nucleotides (5-benzylaminocarbonyl-dU, 5-naphthylmethylaminocarbonyl-dU, 5-tryptaminocarbonyl-dU, and 5-isobutylaminocarbonyl-dU) into the SELEX experiments, the overall success rate increased by ~84% in approximately 1200 proteins analyzed (Gold et al., 2012). The initial modifications of the pyrimidines have been expanded and the method applied to solve large scale analysis of biological samples, such as finding biomarkers for drug development and for diagnosis (Gold et al., 2012). As reported, more than 800 SOMAmers were already selected, meaning that 800 human proteins can be detected simultaneously in a high throughput assay using 15 μl of sample (Gold et al., 2012). According to Gold and collaborators, the new aptamers are so important for technological application that they call them SOMAmers, to distinguish them from the prior literature.

Although aptamers are most frequently defined as DNA or RNA molecules, peptide aptamers are important members of the group. Selection of peptide aptamers depends mainly from a random peptide library (often thioredoxin A is the scaffold protein) and on yeast two-hybridization system (Liu et al., 2013). The importance of the peptide aptamers in the parasitology can be exemplified by a recent report showing the inhibition of DNA damage repair and survival in *T. brucei* by a peptide aptamer mimicking RAD51-binding domain of BRCA2 (Hall et al., 2011). BRCA2, a multifunctional scaffolding protein, is implicated in different cellular processes, including its interaction with DNA recombinase proteins of the RAD51 family. BRCA2 from *T. brucei*, also essential for Variant Surface Proteins switching, interacts with RAD51 through BRC motifs, a 44 amino acid long repeat unit. Interestingly, since *T. cruzi* and *Leishmania* have similar machineries, and *T. brucei* BRCA2 is distinct from mammalian BRCA2, the authors predict the development of an optimized peptide aptamer that will work against the tri trypanosomatids.

DNA and RNA-aptamers applied to studies on trypanosomatids and apicomplexa are discussed below.

### The use of aptamers in the study of parasitic infections

As pointed out, the number of articles in the literature describing the use of aptamers is scarce: while a database search of PubMed reveals almost 3000 publications making use of the term “aptamers” only less than three dozen publications reported the use of aptamers to study pathogens. Of these, most were related to studies on African and American trypanosomes, *Plasmodium* and *Leishmania*, which will herein be focused and are summarized in Table 6 (Goringer, 2012).

**Table 6:** **Summary of the applications of aptamers on protozoan parasite research**.

**Target**	**Library**	**Result**	**References**
*T. cruzi*			
*T. cruzi* ligands for ECM components	2′-F-dU/dc-RNA	50–70% inhibition of epithelial cell invasion by *T. cruzi*. The best inhibition was obtained with aptamers targeted to the laminin receptor	Ulrich et al., 2002
*T. cruzi*	2′-F-dU/dc-RNA	Development of an aptamer-based concentration method for the detection of *T. cruzi* in blood	Nagarkatti et al., 2012
*T. brucei*			
*T. brucei* bloodstream forms	2′-F-dU/dC-RNA	Identification of an aptamer family that binds to a flagellar pocket component. Aptamer internalization through the lysosome pathway	Homann and Goringer, 1999
Preparation of VSG variants	2′-F-dU/dC-RNA	Isolation of aptamers with affinity for many VSGs variants. Binding to live parasites	Lorger et al., 2003
*T. brucei*	2′-NH_2_-dU/dC-RNA	Binding to the flagellar attachment zone	Homann et al., 2006
*Leishmania*			
*L. tarentolae* isolated mitochondria	Reporter RNA	RNA-aptamer base methodology for measuring RNA editing activity in the low femtomole range	Liang and Connell, 2009
*L. tropica*	RNA	Identification of signals for RNA import into the mitochondria	Bhattacharyya et al., 2002
*L. infantum*	DNA	Binding to the surface protein KMP-11	Berberich et al., 1997
*L. infantum*	DNA	Binding to histones (H2A and H3)	Ramos et al., 2007, 2010
*Plasmodium*			
*P. falciparum* PfEMP1	2′-F-dU/dc-RNA	Binding to PfEMP1 exposed at the surface of infected-erythrocytes. Rosette disruption by aptamers	Ghosh et al., 2001, 2002
*P. falciparum* parasitized erythrocyte	DNA	Inhibition of hemozoin formation and parasite growth by heme binding aptamers	Niles et al., 2009

### Studies on kinetoplastidae using aptamers

#### Trypanosoma cruzi

The interaction of *T. cruzi* trypomastigotes with extracellular matrix (ECM) components is vital for the establishment of infection in the vertebrate host (Alves and Colli, 2007). Recognizing the importance of this interaction to the success of *T. cruzi* infection, the SELEX approach was used to evolve aptamers with binding affinities for ECM molecules such as laminin, fibronectin, thrombospondin, and heparan sulfate. Therefore, serum stable RNA aptamers that bind to parasite receptors to ECM were obtained after eight selection rounds, each one involving the incubation of trypomastigotes with a RNA pool (~2 × 10^13^ molecules, consisting of 108 nucleotides with 40-nucleotide randomized region), followed by displacement of the bound molecules with ECM components. One or more iterative rounds were made against *T. cruzi* epimastigotes to eliminate aptamers that bind to targets common to both differentiation forms. Ninety-six clones were sequenced and four classes of RNA aptamers were established based on structural analysis. The selected RNA aptamers bind in the nanomolar range to the parasite receptors for heparan sulfate (40 nM), fibronectin (140 nM), laminin (200 nM), and thrombospondin (400 nM) (Ulrich et al., 2002). Importantly, all four selected aptamers, inhibited *T. cruzi* invasion of epithelial cultured cells by 50–70%, with the best inhibition obtained with the laminin class of aptamers. Although these aptamers confirmed the adhesion of *T. cruzi* to the ECM, the invasion blockage was incomplete, even when all the four classes of aptamers were added together. The existence of other molecules involved in the invasion of host cells by the parasite, an already known complex event (Alves and Colli, 2007) and the binding affinity of the aptamers in the nanomolar range may explain the results. In addition, the possible clearance of the aptamers from the surface of the parasite by shedding has to be considered. Remarkably, GPI-anchored surface glycoproteins from the Tc85 group that bind to laminin (Giordano et al., 1999) are shed as membrane vesicles (Torrecilhas et al., 2012), with a 3.5 h half-life (Gonçalves et al., 1991). The data demonstrate that the SELEX technique can be employed to isolate aptamers against parasite targets, which may be useful in inhibiting parasite invasion. However, aptamers that recognize other molecules may be included, as for example molecules that are also shed and are known to enhance parasite invasion, as gp85/transialidase (Tonelli et al., 2011; Rubin and Schenkman, 2012) to improve the possibility of finding therapeutic approaches through aptamers.

Due to migration, approximately 390,000 humans infected with *T. cruzi* were detected in the United States (~75% of the cases), Europe, western Pacific, Canada, Japan and Australia (Coura and Vinas, 2010). In this scenario, since blood transfusion is one way of *T. cruzi* transmission, methods with high sensitivity and specificity for the parasite detection are crucial. Most of the methodologies developed are based on antibodies (ELISA assays) or live parasites (PCR). However, false negative results can be obtained in both cases, due to low amount of antibodies (as in the initial phase of the disease) or to few circulating parasites (as in the asymptomatic cases or chronic phase of the disease). Moreover, detection of antibodies is not a good parameter of cure for Chagas' disease, since the antibodies persist in circulation for a certain period of time, even after the parasite killing, in addition to cross-reactivity problems among the trypanosomatids parasites.

Looking for the improvement of a diagnostic method based on live parasites, an aptamer-based concentration of *T. cruzi* in blood was recently reported (Nagarkatti et al., 2012). Using SELEX strategy, serum stable RNA aptamers that bound to live trypomastigotes with high affinity (8–25 nM ranges) were selected. One aptamer (Apt68) bound specifically to trypomastigotes, but not to epimastigotes or other related trypanosomatids (*L. donovani* and *T. brucei*) with high affinity (*K*_*d*_ ~7.6 nM). It was also shown that Apt68 immobilized on a solid phase was able to capture and aggregate trypomastigotes from different strains. Moreover, using a magnet, trypomastigotes aggregated to Apt68-coated paramagnetic beads could be purified from the blood and detected by real-time PCR assay, even at concentrations as lower as five trypomastigotes/15 ml. The purification step of the parasite prior to DNA extraction presents technical advantages in relation to the direct extraction from the whole blood, in addition to the volume reduction of the blood sample employed.

#### African trypanosomes

Aptamers against the infective bloodstream forms of *T. brucei* were selected by incubating the parasite with a RNA library (2 × 10^15^ unique sequences) (Homann and Goringer, 1999). After 12 cycles of selection, 53 clones were sequenced, resulting in the identification of three structural families of aptamers, one of which being further characterized. The aptamer 2–16 RNA was specific for the bloodstream stage, since it did not react with the insect stage of the parasite, and bound with high affinity (*K*_*d*_ ~60 nM) to a 42 kDa protein located within the flagellar pocket of the parasite. Moreover, the aptamer also bound to two other *T. brucei* strains. In order to be employed *in vivo*, the aptamer 2–16 was chemically modified, retaining the same properties as the original unmodified molecule, as well as a good stability *in vivo* (half-life in serum of 3.4 days) (Adler et al., 2008). Aptamers that recognized the flagellar attachment zone with high affinity (*K*_*d*_ = 70 ± 15 nM) were also selected against the bloodstream stage of *T. brucei* (Homann et al., 2006). The half-life was determined as more than 30 h, showing a good stability in serum, an important characteristic for using the aptamer as a therapeutic device.

As pointed out before, bloodstream forms of *T. brucei* evade the host immune system by switching temporarily the single VSG that covers the surface of the parasites (antigenic variation). Despite the low identity of the amino acid composition among the VSG repertoire, the presence of similar tertiary structures, hidden from the antibody attack, was the rationale for selecting aptamers against the conserved structures (Lorger et al., 2003). For the selection, a homogeneous preparation of VSG (variant 117) was incubated with a combinatorial library of 2 × 10^14^ unique RNA sequences. After three rounds of selection, the aptamers were incubated with live parasites expressing the same VSG variant to remove RNAs that did not recognize the native membrane-bound protein. Finally, the same scheme was employed using a different VSG (variant 221) and parasites expressing VSG 221. As a result, RNA aptamers common to both VSG variants were obtained. After round nine, 60 individual clones were sequenced and placed into three groups, according to sequence motifs (76% in group I) in addition to orphan RNAs (6%). Aptamers from the three groups bound to the VSGs in the nanomolar or subnanomolar concentration range (*K*_*d*_ = 0.16 ± 0.02 nM for clone nine), recognized other VSG variants from *T. brucei*, as well as from *T. congolense* and showed good serum stability (15 h). The biotin-labeled aptamers/fluorescent-conjugated streptavidin methodology demonstrated binding of the three groups of aptamers to the whole surface of live *T. brucei* expressing different VSG variants. Also, the biotin-labeled aptamers bound to the surface of the trypanosome and detected by anti-biotin antibodies could be used to drive immunoglobulins to the surface of the parasite.

#### Leishmania tarentolae

RNA editing, a molecular process found in trypanosomatids, regulates gene expression in their sole mitochondrion. It consists in the insertion and deletion of uridylates in mitochondrial mRNAs guided by small RNAs. The process occurs in a multi-protein complex (editosome), with endonuclease, uridylate transferase, uridylate-specific 3′exoribonuclease and RNA ligase being the four major enzymes involved. Incorrect function of editosome leads to parasite death, as shown for *T. brucei* (Schnaufer et al., 2001; Tarun et al., 2008). Although editosomes are common to other Kinetoplastidae parasites, their protein content may not be identical, as shown for example for two new proteins from *T. brucei* (KREPB9 and KREPB10), which are associated with the editing activity of the endonuclease. Both proteins are absent in *Leishmania* and KREP 10 is absent in *T. vivax* (Lerch et al., 2012). RNA editing is an attractive target for chemotherapy, as pointed out by the abundant literature in the field (Niemann et al., 2011).

The identification and exact function of each component of the editosome is then essential and sensitive assays to detect editing activity have been proposed. Among them, a RNA aptamer-based methodology was developed using a mitochondrial population from *L. tarentolae* as an initial source (Liang and Connell, 2009). The method detects the editing activity by an electrochemiluminescent signal generated by an editing responsive conformational change within a ruthenium labeled RNA reporter. It is claimed that the method detects the edited product in the femtomole range and can be performed in small volumes (12 μl in 384-well microtiter plate) making it suitable for high-throughput screening.

#### Leishmania tropica

The SELEX method was employed to identify the import signals of cytoplasmatic RNA by mitochondrial receptors. After four rounds of SELEX, the pool of aptamers was further selected by its capacity to be efficiently imported by the mitochondria. Four aptamers were studied in more detail and classified in two types by their efficiency in crossing the inner mitochondrial membrane (type I: A and D arm homologues; type II–V-T homologues, a new putative import signal). Furthermore, considering the efficiencies of aptamers transport into the mitochondria, cooperative and antagonistic interactions among them have been reported, which may be important for the regulation of RNA import, as suggested by the authors (Bhattacharyya et al., 2002).

#### Leishmania infantum

KMP-11 is a surface protein associated with Kinetoplastidae parasites cytoskeleton, such as *T. cruzi*,* T. brucei*,* L. donovani*, and *L. infantum* and differentially expressed during the parasite life cycle (Tolson et al., 1994; Stebeck et al., 1995; Berberich et al., 1997). A high antibody response against KMP-11 during natural infection of *L. infantum* was reported (Berberich et al., 1997), as well as the stimulation of T-lymphocytes proliferation (Tolson et al., 1994). These characteristics raised the possibility that KMP-11 may be an important tool against infection by *Leishmania*. DNA aptamers were then selected against KMP-11 protein and after a 10-round cycle, aptamers that specifically recognized KMP-11 were selected (Moreno et al., 2003) and are awaiting further characterization.

Although highly conserved among different eukaryotes, histones from trypanosomatids show sequence divergences in the amino- and carboxy-terminal domains. High affinity and specific DNA aptamers were then selected against *L. infantum* H2A (Ramos et al., 2007) and H3 (Ramos et al., 2010).

### Studies on apicomplexa using aptamers

#### Plasmodium

During erythrocyte infection *P. falciparum* secretes proteins to the surface of the RBCs associated with cytoadherence of the infected erythrocytes to the endothelium of blood vessels or to other non-infected erythrocytes, monocytes, and platelets, leading to rosette formation, a phenotype associated with virulence. The presence of infected erythrocytes in microvessels associated with host responses to the sequestered erythrocytes are centrally involved in the disease pathology. Moreover, 48 cycles of invasion and development inside erythrocytes are made possible by the cytoadherence mechanism while the parasite circulates in the bloodstream. Cytoadherence and rosetting are associated with PfEMP-1 protein (parasite-derived erythrocyte membrane protein) that is exposed on the erythrocyte surface and binds to a different number of human cell receptors, such as heparan and chondroitin sulfates, ICAM-1, CD36 (Fairhurst et al., 2012). A good strategy to minimize malaria mislays is to raise ligands for a more conserved region of PfRMP-1 responsible for adhesion, such as the DBL1α, a rosette forming domain. Using a combinatorial library of 5 × 10^14^ unique RNA sequences and eight rounds of selection against DBL1α, 85 clones were sequenced and analyzed (Barfod et al., 2009). Two aptamers that bind to PfEMP1 on the surface of infected erythrocytes decrease the rosette formation by 35% at 33 nM concentration and by 100% at 38 nM, pointing out to their potential use as candidates for severe malaria therapy (Barfod et al., 2009).

Hemoglobin digestion in *Plasmodium* occurs in acidic vacuoles, as a result of an extensive uptake of erythrocytic cytoplasm by mid ring and mid trophozoite stages, both early intraerythrocytic stages of the parasite (Bakar et al., 2010). The digestion results in the production of globin, a source of amino acids, and free heme molecules (Fe^2+^-protoporphyrin IX), which are toxic to the parasite. The oxidation of the heme group results in Fe^3+^-protoporphyrin IX, which precipitates inside the vacuoles as crystals known as hemozoin (malaria pigment) (Weissbuch and Leiserowitz, 2008).

Anti-malarial drugs extensively used in clinic, as chloroquine or artemisinin act during the degradation of hemoglobin and subsequent hemozoin formation (Weissbuch and Leiserowitz, 2008). Artemisinin, for instance, delays hemoglobin uptake and a product of the hemoglobin hydrolysis potentiates its activity (Klonis et al., 2011). The pathway for hemoglobin degradation is then a suitable target for the development of new drugs and aptamers are good candidates. Heme binding DNA aptamers previously selected *in vitro* by SELEX carried out at pH 7–8 (Li et al., 1996; Okazawa et al., 2000) were employed to interfere with the heme-detoxification in *P. falciparum*. The selected aptamers bind equally to heme at acidic pH, the environmental condition of the vacuolar structures responsible for hemoglobin digestion, and inhibit hemozoin formation *in vitro*. Two of the heme-binding aptamers were used to verify their effect on parasite growth. The erythrocytes were first loaded with the aptamers using hypotonic lysis and resealing conditions, reaching the level of 65–85% of aptamers-loaded cells. Parasite growth in the preloaded-heme binding aptamers cells was significantly reduced after 72 h in culture, when compared to the controls (Niles et al., 2009). The aptamers, as suggested, may be useful tools for elucidation and manipulation of pathways important for the parasite survival.

## Concluding remarks

Phage display and SELEX are powerful methodologies to be employed in a variety of systems to approach different questions, from the understanding of cell biology to biotechnology. Both techniques share important characteristics, such as the possibility to use large oligonucleotide-based or phage display peptide libraries to screen almost any target, including the whole living cell or animal models. Chemical modification of aptamers and peptides poses an additional advantage over the phage display as it allows more stability in blood for therapeutic use. Particularly, aptamers seem to lack immunogenicity, which, associated with high affinity and specificity to their targets may easily substitute antibodies for therapeutic purposes, although a fast clearance of the aptamers have been shown in the literature. Besides its use directly to neutralize its target, aptamers also can be used as agents to deliver nanoparticles loaded with specific therapeutic drugs or iRNA (Liu et al., 2013). Blood-living or transiently blood-living parasites, as *T. brucei*, *T. cruzi*, or *Plasmodium* are good targets for this kind of approach. The same rational can be easily applied for any parasite-induced modification of the host cell surface, such as the knob-like structures in *Plasmodium*-infected erythrocytes. However, its application will be much more complex when parasites that live into the host cell cytoplasm (such as *T. cruzi*) or inside vacuoles (such as *Leishmania*) are considered. Indeed, as the advantages of target therapy become more apparent, the use of phage display and aptamers to retrieve for disease-related antigens are receiving increased attention. Additionally, aptamers-carrying iRNA may have diverse applications, as helping the understanding of the role of the innumerous proteins involved in the parasite–host cell interaction or inhibiting the expression of proteins from parasites, which possess the iRNA machinery. Besides its use on parasite–host interactions, phage display technology may be engineered for a particular functional activity as, for example, to display human antibodies with high affinity and specificity for particular antigens in disease. In humans, phage antibody libraries made from donors who naturally mount an immune response (with viral infections, bearing tumors, or with autoimmune disease) is being used not only to investigate the humoral response in disease but also with clinical purposes (Hoogenboom and Chames, 2000). These would be an interesting approach as therapy against parasitic diseases.

The application of aptamers for molecular imaging is being used in clinics for the evaluation of several diseases (Cibiel et al., 2012) with the great advantage of not being an invasive methodology and may be helpfully applied also in parasitology. An exciting use of the technique for cell biology is the imaging of cellular metabolites with RNA-based sensors recently described, where the intracellular levels of ADP and S-adenosylmethionine were measured (Paige et al., 2012). Since RNA aptamers to any molecule can be rapidly selected, a rapid expansion of the methodology to other small molecules is envisaged.

As shown by the low number of reports in the literature, it is surprising that these methodologies were not, as yet, employed more often in the study of the complex phenomenon of host–parasite interaction, with the aim to develop much needed diagnostic and therapeutic tools to combat infections caused by protozoan parasites. Notwithstanding, considering the constant and fast improvement of both technologies and their large applicability to different biological problems, it is expected that phage display and SELEX will make, in a near future, significant contributions to the field of protozoan-borne diseases.

### Conflict of interest statement

The authors declare that the research was conducted in the absence of any commercial or financial relationships that could be construed as a potential conflict of interest.
